# Competitive Inhibitors Unveil Structure/Function Relationships in Human D-Amino Acid Oxidase

**DOI:** 10.3389/fmolb.2017.00080

**Published:** 2017-11-27

**Authors:** Gianluca Molla

**Affiliations:** ^1^Department of Biotechnology and Life Sciences, University of Insubria, Varese, Italy; ^2^The Protein Factory Research Center, Politecnico of Milano and University of Insubria, Milan, Italy

**Keywords:** D-amino acid oxidase, D-serine, inhibitor, schizophrenia, structure, neurotransmission, flavoprotein, drug design

## Abstract

D-amino acid oxidase (DAAO) catalyzes the oxidative deamination of several neutral D-amino acids and is the enzyme mainly responsible (together with serine racemase) for degrading D-serine (D-Ser) in the central nervous system of mammals. This D-amino acid, which binds the coagonist site of the N-methyl-D-aspartate receptor, is thus a key neuromodulator of glutamatergic neurotransmission. Altered D-Ser metabolism results in several pathological conditions (e.g., amylotrophic lateral sclerosis or schizophrenia, SZ) for which effective “broad spectrum” pharmaceutical drugs are not yet available. In particular, the correlation between reduced D-Ser concentration and SZ led to a renaissance of biochemical interest in human DAAO (hDAAO). In the last 10 years, public and corporate research laboratories undertook huge efforts to study the structural, enzymatic, and physiological properties of the human flavoenzyme and to identify novel effective inhibitors which, acting as pharmaceutical drugs, could decrease hDAAO activity, thus restoring the physiological concentration of D-Ser. Although, none of the identified hDAAO inhibitors has reached the market yet, from a biochemical point of view, these compounds turned out to be invaluable for gaining a detailed understanding of the structure/function relationships at the molecular level in the mammalian DAAO, in particular of the interaction between ligand and the enzyme. This detailed knowledge, together with several recent studies concerning the interaction of the human enzyme with other protein regulative partners, its subcellular localization, and *in vivo* degradation, contributed to gaining comprehensive knowledge of the structure, function, and physiopathological role of this important human enzyme.

## Introduction

**Figure 1:**
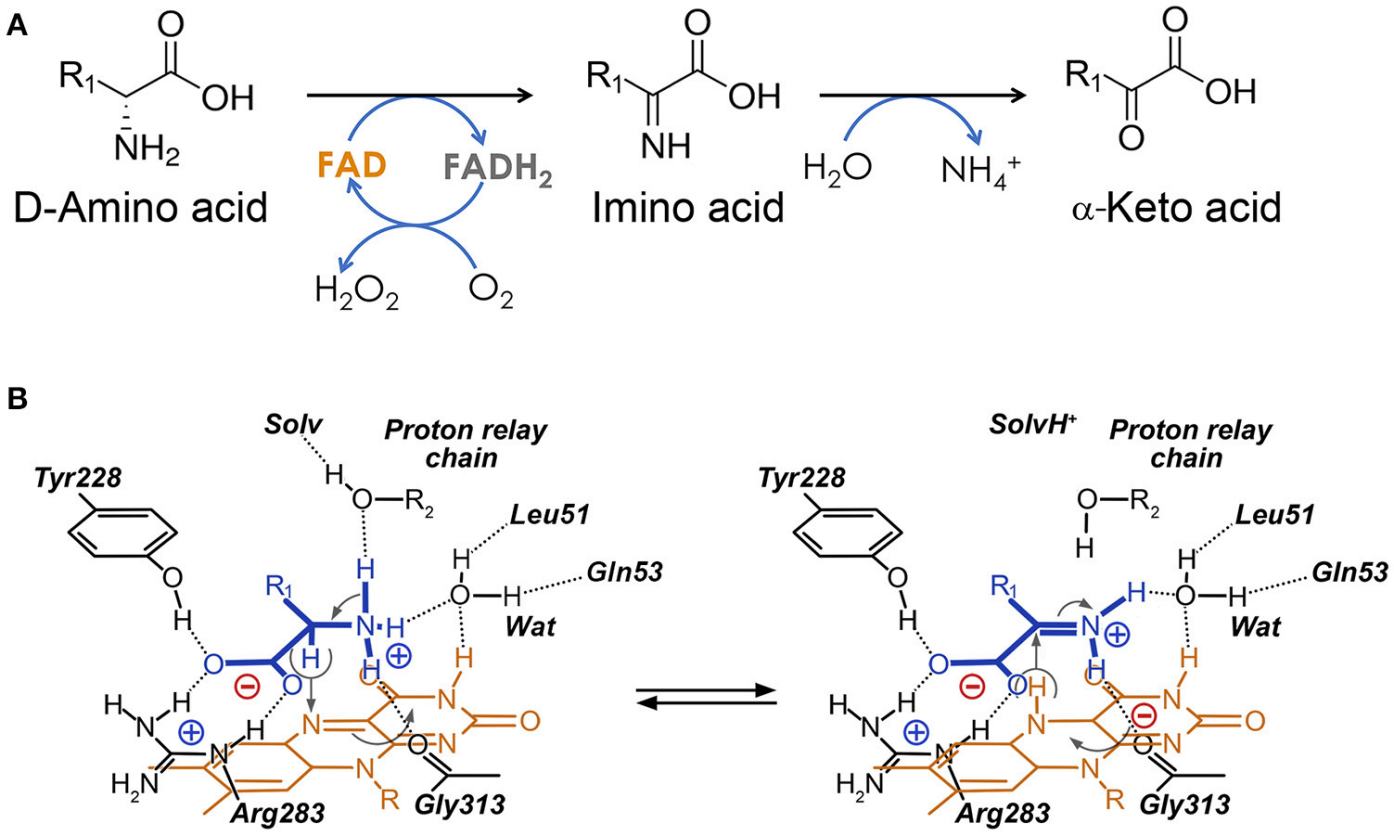
Reaction catalyzed by human D-amino acid oxidase. **(A)** Scheme of the reaction. R_1_ represents the side chain of the substrate. **(B)** Schematic representation of the hydride transfer mechanisms. The D-amino acid is bound in its zwitterionic form. Substrate is colored in blue. H-bonds are represented as dotted lines. Arrows, indicating relocation of electrons during the reaction, are represented in gray. Wat: putative active-site water molecule. R_1_: side chain of the substrate. The first H acceptor of the proposed proton relay chain (at pH < 8) has not yet been identified: it could be an active-site water molecule (R_2_ = H) or the hydroxyl of a protein residues (R_2_ = protein).

D-amino acid oxidase (E.C. 1.4.3.3, DAAO), originally designated as “the new yellow enzyme,” was the second flavoprotein to be discovered at the beginning of the last century and it is considered the prototype of flavin-containing oxidases (Curti et al., 1992). DAAO catalyzes the oxidative deamination of D-amino acids to the corresponding imino acids which, in aqueous solutions, spontaneously hydrolyze, yielding the corresponding α-keto acids and ammonia; the reduced FAD cofactor is rapidly reoxidized by molecular oxygen to produce H_2_O_2_ (Figure 1A). Although the gene coding for the human enzyme was cloned in the late 1980s (Momoi et al., 1988), most of the functional and structural studies on mammalian DAAOs were performed on the enzyme purified from pig kidney (Mattevi et al., 1996; Pilone, 2000; Pollegioni et al., 2007).

Notwithstanding the fact that human DAAO (hDAAO) activity was detected for the first time in the human brain in 1966 (Neims et al., 1966), the function of this flavoenzyme in the mammalian central nervous system (CNS) remained elusive until 1992 when the development of novel sensitive analytical techniques allowed the detection in rat brain of substantial amounts of free D-Ser (its physiological substrate in CNS) (Hashimoto et al., 1992). These findings were subsequently further supported by the observed close correlation of the distribution of endogenous D-Ser with that of the N-methyl-D-aspartate receptors (Hashimoto et al., 1993; Schell et al., 1995). This D-amino acid is a key neuromodulator of glutamatergic neurotransmission: it is a coagonist of the N-methyl-D-aspartate type glutamate receptor to which it binds at the “strychnine-insensitive glycine modulatory site” (NR1 subunit) (Mothet et al., 2000). D-Ser is also a ligand of the δ2 glutamate receptor (Kakegawa et al., 2011). In brain, D-Ser is synthesized from L-Ser by the PLP-dependent enzyme serine racemase (EC 5.1.1.18) and is degraded by the serine racemase itself and by hDAAO (Foltyn et al., 2005; Pollegioni and Sacchi, 2010). An anomalous increase in D-Ser levels has been correlated with acute or chronic neurodegenerative diseases (e.g., stroke, epilepsy, amyotrophic lateral sclerosis, Parkinson's disease, Alzheimer's disease, and Huntington's disease), while an anomalous decrease in concentration has been correlated with severe psychiatric disorders (e.g., schizophrenia and bipolar disorders) (Sacchi et al., 2012). A decrease in the concentration of D-Ser was also observed in rats and mice during aging due to a lower expression of serine racemase (while the expression of DAAO was unchanged) (Billard, 2015).

Up to now the search for a “broad spectrum” pharmaceutical drug effective against SZ has been disappointing; drugs such as chlorpromazine (CPZ, a dopamine D2 receptor antagonist introduced in the 1950s), clozapine, or perphenazine show several serious side effects (Abbott, 2010). In 2002, a landmark genetic analysis by Chumakov et al. (2002), confirmed by several following association studies, linked the genes coding for hDAAO and its putative regulatory interactor pLG72 to SZ susceptibility.

From a biochemical point of view, the correlation between increased hDAAO activity and abnormally low levels of D-Ser (that, in turn, could result in hypofunctional glutamatergic neurotransmission) suggests new strategies for the pharmacological treatment of the heterogeneous symptoms of this pathology. This physiopathological biochemical hypothesis led to a renaissance of biochemical interest in hDAAO in the last 10 years; its structure and biochemical properties were investigated in detail in 2006 (Kawazoe et al., 2006; Molla et al., 2006), and hundreds of new hDAAO inhibitors have since been discovered. Importantly, the 3D structure of hDAAO in complex with 15 different compounds has been solved. This huge amount of data has given us a detailed understanding of the structure/function relationships in this enzyme.

## Physiological role and tissue distribution of human D-amino acid oxidase

**Figure 2:**
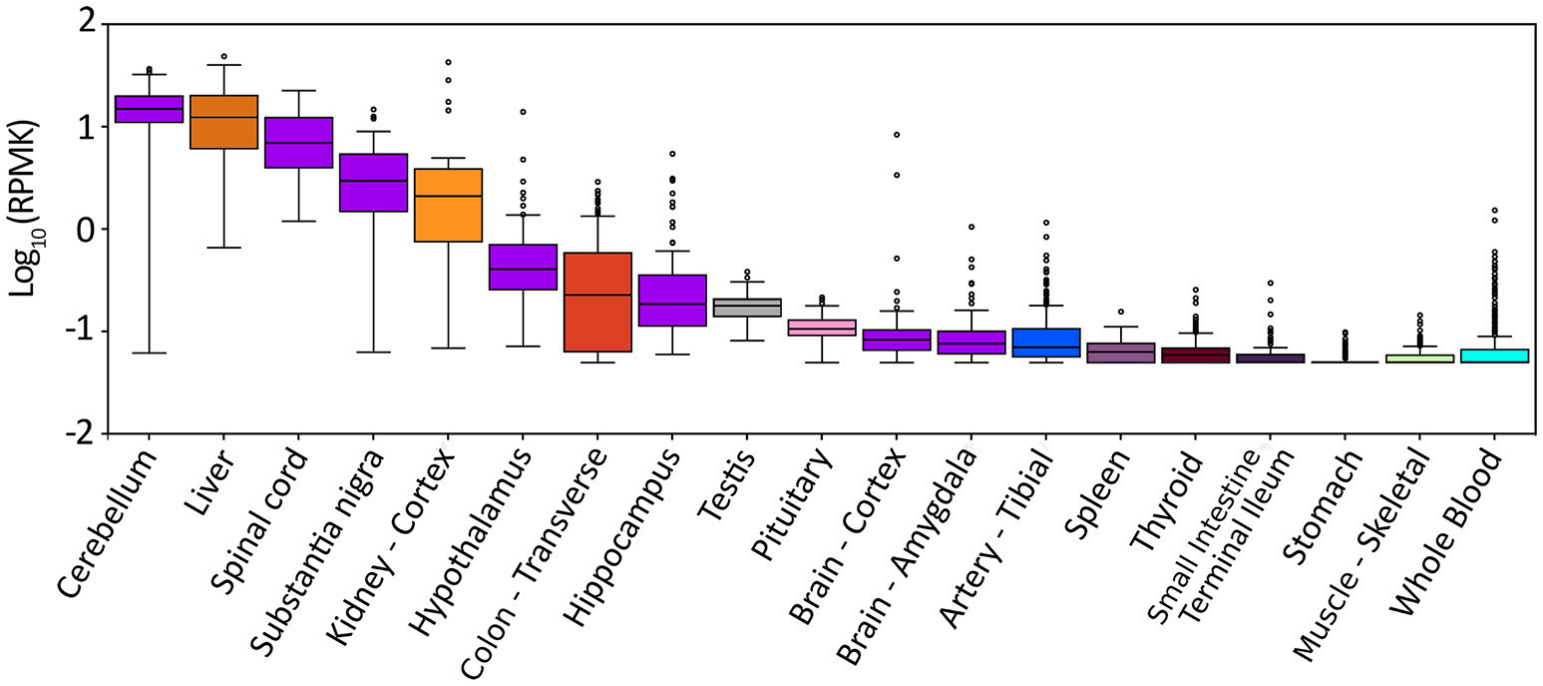
Expression pattern of *DAO* gene in different tissues. Box plot of expression levels of *DAO* gene in different human tissues. Values are shown in RPKM (reads per kilobase of transcript per million mapped reads), calculated from a gene model with isoforms collapsed to a single gene. No other normalization steps have been applied. Box plots are shown as median and 25th and 75th percentiles; points are displayed as outliers if they are above or below 1.5 times the interquartile range. The data presented in this plot were obtained from the Genotype-Tissue Expression (GTEx) Project Portal, dbGaP accession number phs000424.v6.p1 on 09/04/2017 (https://www.gtexportal.org/home/gene/DAO).

The gene for hDAAO (*DAO*) is encoded on chromosome 12 (region 12q24) (Konno, 2001). Based on the GTEx Analysis (Release V6p, dbGaP Accession phs000424.v6.p1), hDAAO is prevalently expressed in three organs: liver, kidney, and brain (Figure 2). The expression pattern is tightly intertwined with the physiological functions of the enzyme: in liver and kidney (and, to a lesser extent, in the urinary apparatus and in colon), hDAAO is involved in detoxification and elimination of D-amino acids originating from endogenous racemization or presumably from the diet. hDAAO is also expressed in neutrophilic leukocytes, where the protein, usually localized close to the cell surface, is internalized during phagocytosis (Robinson et al., 1978). It has been hypothesized that hDAAO (together with the enzyme myeloperoxidase) may exert a protective effect against bacterial infection. hDAAO is able to utilize bacterial lysate, which contains D-Ala derived from peptidoglycan, to generate hydrogen peroxide, which is toxic for bacteria. As a matter of fact, DAAO knockout mice are more susceptible to *S. aureus* infection than wild-type mice (Nakamura et al., 2012).

In the CNS, hDAAO expression is complementary to D-Ser concentration: the enzyme is mainly expressed in the cerebellum (in particular in cerebellar Bergmann glia) and brainstem (where the D-Ser concentration is low) while its expression is lower in the forebrain (where the overall D-Ser concentration is higher) (Figure 2; Horiike et al., 1994). The actual fine distribution of hDAAO in the CNS is still controversial due to differences between the patterns of gene expression and enzyme activity. Interestingly, in different brain regions, hDAAO is expressed in different cell types, namely, glial cells, neurons, and astrocytes. Controlling the availability of the neuromodulator D-Ser through its degradation represents the proposed role for astrocytic hDAAO in the gray matter while, in white matter, the effective clearance of D-Ser by astrocytic hDAAO could prevent excitotoxicity due to an overload of glutamate (Sacchi et al., 2011).

Concerning the subcellular localization of hDAAO, the enzyme largely localizes in peroxisomes because of the presence of the C-terminal peroxisomal PTS1 targeting sequence: the tripeptide Ser-His-Leu (Pollegioni et al., 2007). This opens the question concerning how (and when) the human flavoenzyme could “encounter” its physiological substrate D-Ser inside the cell. However, it must be noted that, in brain cells, hDAAO does not show an exclusively peroxisomal localization. In U87 human glioblastoma cells, the recombinant hDAAO shows a spatiotemporal distribution: 24 h after transient transfection, a fraction of folded and active hDAAO is largely diffused in the cytosol, where it can interact with different proteins such as pLG72, the PTS1-receptor Pex5p, or the protein bassoon in neurons (Popiolek et al., 2011; Sacchi et al., 2011). Subcellular localization of hDAAO also affects the pathway and rate of degradation. In U87 cells, the degradation of the peroxisomal fraction of hDAAO is linked to the pexophagy process (i.e., the “physiological turnover” of the peroxisome by autophagy), resulting in a half-life that is quite long (t_1/2_ ~ 60 h). On the other hand, cytosolic hDAAO (characterized by a significantly shortened half-life) is essentially targeted to the ubiquitin proteasome system (Cappelletti et al., 2014).

## Molecular evolution and overall structure

**Figure 3:**
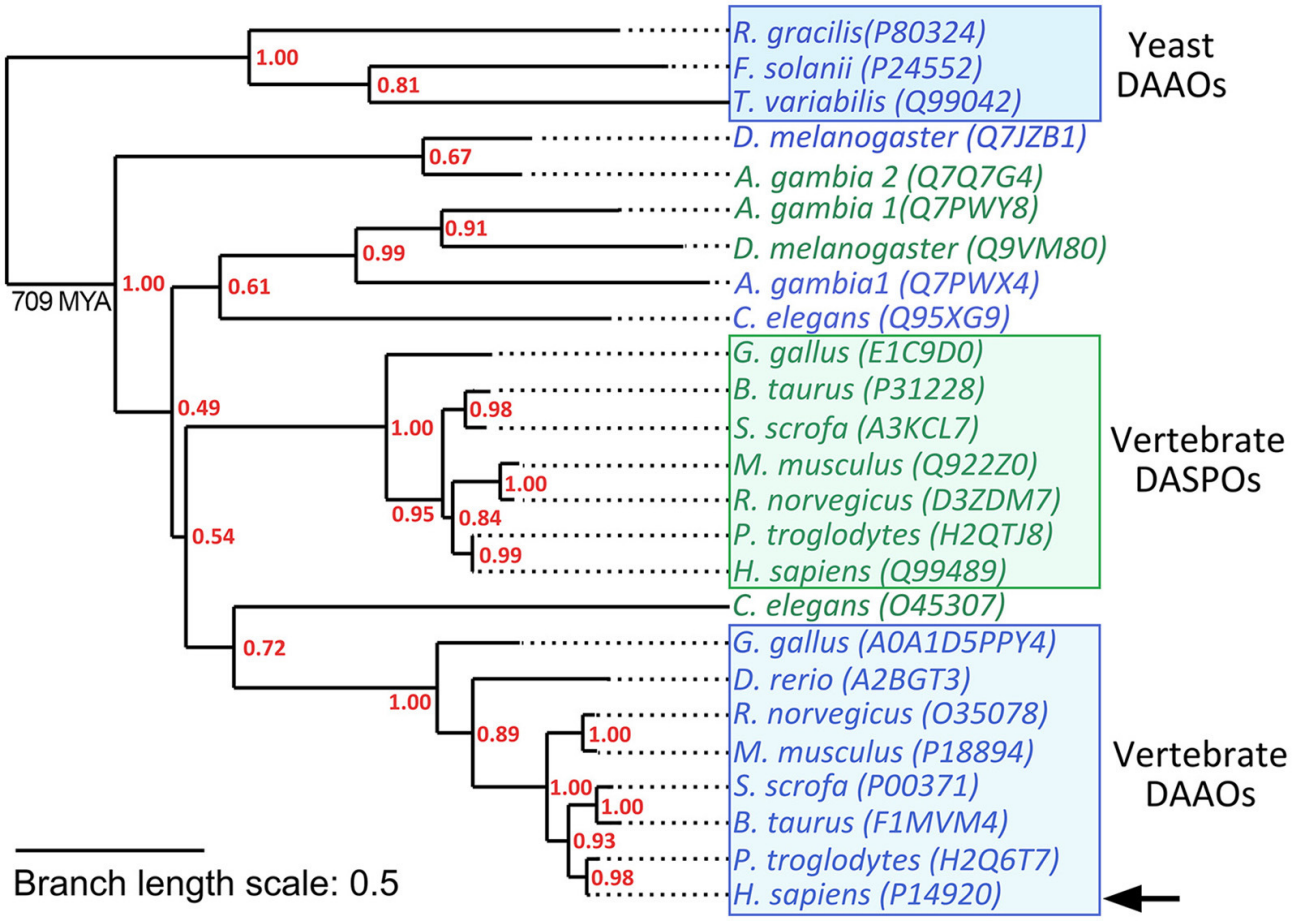
Schematic phylogenetic tree of DAAOs and DASPOs from selected model species. Yeast DAAOs represent the outgroup allowing the phylogenetic tree rooting. Selected sequences were aligned using Clustal Omega Server. Alignment was used to calculate a maximum-likelihood phylogenetic tree using PhyML 3.0 server (Guindon et al., 2010). hDAAO is indicated by an arrow. Sequences were identified by their UniProtKB accession number. DAAO sequences are in blue while DASPO sequences are in green. Supporting values for each node are represented in red. Branch length scale units represent the nucleotide substitutions per site.

hDAAO belongs to the amino acid oxidase family of flavoproteins. Members of this large protein family share a common ancestor which acquired specific structural and functional features in different organisms during evolution. This allowed integration of these enzymes into different metabolic pathways to fulfill distinct physiological functions. In vertebrates, two paralogous amino acid oxidases (which possess a different substrate specificity) are expressed, i.e, the DAAO and D-aspartate oxidase (DASPO). The genes coding for these two proteins originated via a gene duplication event that can be roughly dated to after the divergence of *Ascomycota* from *Metazoa* (~1198 MYA) and before the divergence of *Protostomia* from *Deuterostomia* (~709 MYA) (Hedges et al., 2015). As a consequence, insects and vertebrates possess both DAAO and DASPO while yeast species do not produce DASPO with the sole exception of *Vanrija humicola* (*Cryptococcus humicola*) which expresses a flavoprotein oxidase active on D-Asp. Interestingly this latter enzyme is more closely related to fungal DAAOs than to mammalian DASPOs (Takahashi et al., 2004; Figure 3).

**Figure 4:**
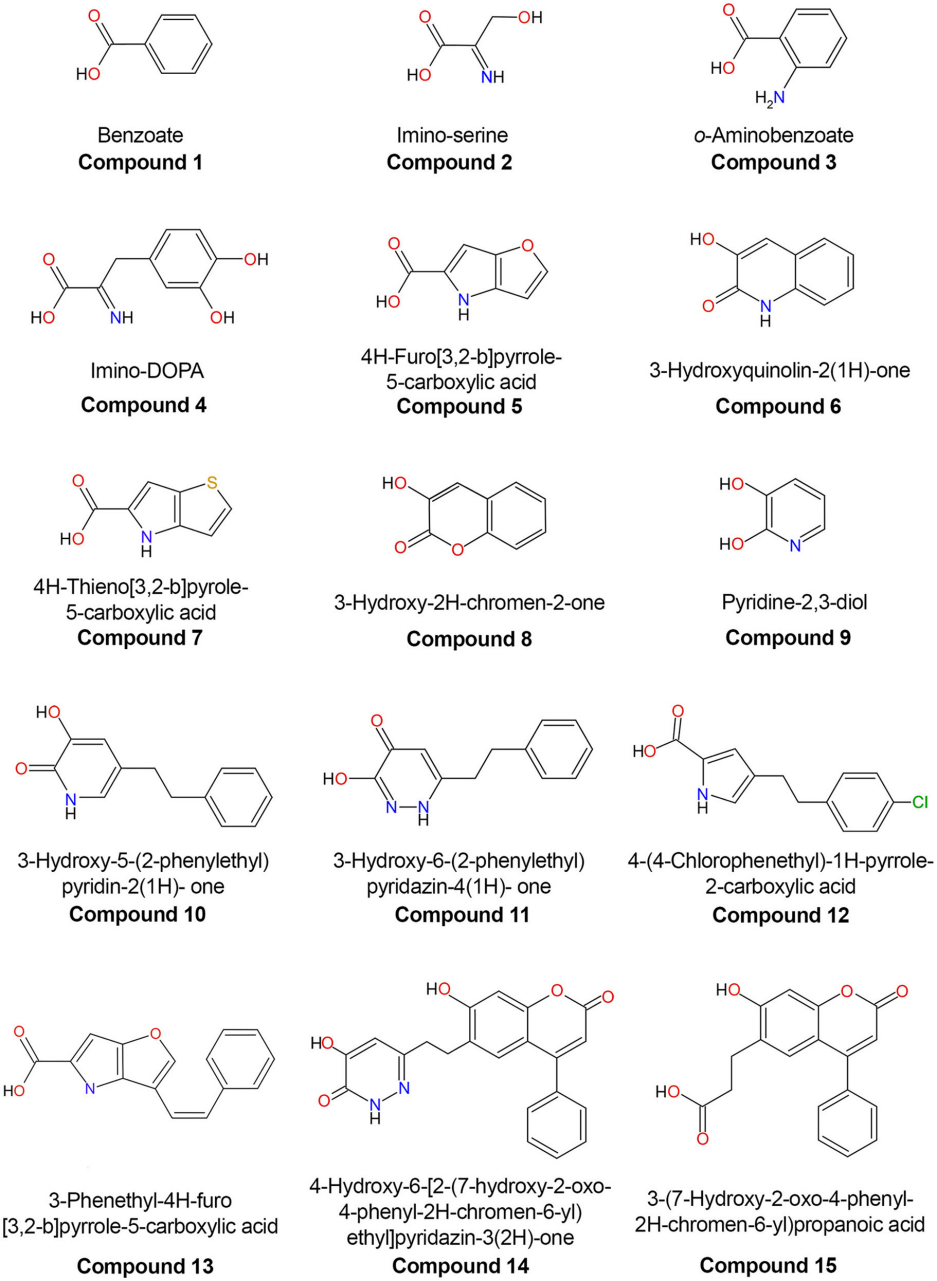
Chemical structures of human D-amino acid oxidase ligands reported in Table 1.

The first three-dimensional structure of the human DAAO was solved in 2006, 10 years after the structure of the homologous protein from pig kidney was determined (Mattevi et al., 1996; Kawazoe et al., 2006). The potential role of the human enzyme as a target for pharmaceutical drugs against SZ spurred the structural investigation: up to now, 16 different experimental 3D structures of the wild-type hDAAO in the free form and in complex with different ligands have been deposited in the RCSB Protein Data Bank (Figure 4; Table 1).

**Table 1:** Experimental 3D structures of human DAAO available in the Protein Data Bank.

**Compound**	**PDB entry**	**Release year**	**Res. (Å)**	**Ligand name**	**References**
	2E48	2007	2.90	No ligand	Kawazoe et al., 2007b
1	2DU8	2006	2.50	Benzoate	Kawazoe et al., 2006
2	2E49	2007	3.20	Imino-serine	Kawazoe et al., 2007b
3	2E4A	2007	2.60	*o*-Aminobenzoate	Kawazoe et al., 2007b
4	2E82	2007	2.70	Imino-DOPA	Kawazoe et al., 2007b
5	3CUK	2008	2.49	4H-Furo[3,2-b]pyrrole-5-carboxylic acid	Sparey et al., 2008
6	3G3E	2009	2.20	3-Hydroxyquinolin-2(1H)-one	Duplantier et al., 2009
7	3ZNN	2013	1.90	4H-Thieno[3,2-b]pyrole-5-carboxylic acid	Hopkins et al., 2013a
8	3ZNP	2013	2.40	3-Hydroxy-2H-chromen-2-one	Hopkins et al., 2013a
9	3W4I	2013	2.50	Pyridine-2,3-diol	Hondo et al., 2013
10	3W4J	2013	2.74	3-Hydroxy-5-(2-phenylethyl)pyridin-2(1H)-one	Hondo et al., 2013
11	3W4K	2013	2.86	3-Hydroxy-6-(2-phenylethyl)pyridazin-4(1H)-one	Hondo et al., 2013
12	3ZNO	2013	2.30	4-(4-Chlorophenethyl)-1H-pyrrole-2-carboxylic acid	Hopkins et al., 2013a
13	3ZNQ	2013	2.75	3-Phenethyl-4H-furo[3,2-b]pyrrole-5-carboxylic acid	Hopkins et al., 2013a
14	4QFC	2014	2.40	4-Hydroxy-6-[2-(7-hydroxy-2-oxo-4-phenyl-2H-chromen-6-yl)ethyl]pyridazin-3(2H)-one	Terry-Lorenzo et al., 2014
15	4QFD	2014	2.85	3-(7-Hydroxy-2-oxo-4-phenyl-2H-chromen-6-yl)propanoic acid	Terry-Lorenzo et al., 2014

*Structures of ligands are reported in Figure 4*.

**Figure 5:**
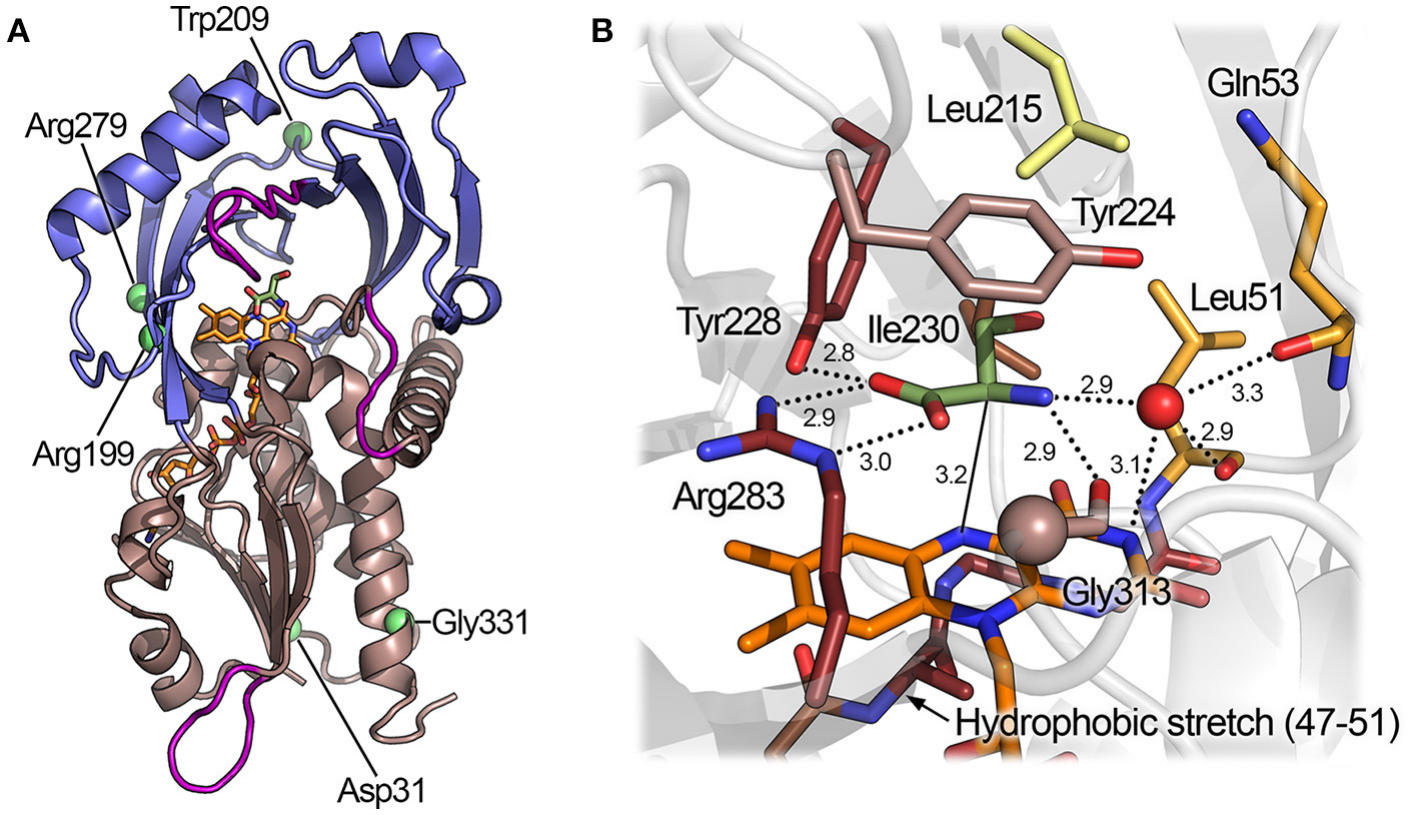
Three-dimensional structure of hDAAO. **(A)** Schematic view of the spatial arrangement of protein domains in hDAAO (PDB code 2e49) (Kawazoe et al., 2007b). The FAD binding domain is in dark salmon color and the substrate binding domain is in blue. Regions showing the highest conformational diversity are in purple; α-helices, β-strands, and coils are drawn in cartoon representation. **(B)** Detail of the active site. The substrate D-Ser was modeled based on the structure of the enzyme in complex with imino-serine. Residues are colored according to their ConSurf score from darker (most conserved) to lighter (less conserved) color. Gly313 αC is represented as a sphere. The “putative” active-site water molecule is in red (see text). The FAD cofactor (orange) and the ligand (green) are shown in stick form. The H-bonds are represented by dotted lines while the distance between the α-H of the substrate and the N(5) of FAD is depicted with a continuous line.

The hDAAO is always present in solution as a homodimer: each monomer, formed by 347 residues, binds one FAD molecule in a non-covalent fashion, yielding a 40.3-kDa complex. The overall tertiary structure of hDAAO consists of two interconnected domains: an FAD-binding domain (FBD), composed of residues 1–87, 140–195, and 286–347, and a substrate-binding domain (SBD), composed of residues 88–139 and 196–285 (Figure 5A; Kawazoe et al., 2006). Both domains possess an α/β architecture in which the large, central, twisted β-sheet is sandwiched between α-helices on both sides. In the FBD, the parallel β-sheet contains 6 β-strands; based on the primary and tertiary structure conservation of this domain (which possesses the canonical Rossmann fold motif), DAAO can be classified in the second glutathione reductase subfamily of flavoproteins (Dym and Eisenberg, 2001). In the SBD, the (mainly) antiparallel β-sheet contains 8 β-strands and forms the active site roof and a large part of the dimerization interface. Global superimposition of the available 3D structures of hDAAO reveals that the conformational flexibility of the protein is generally low, with an overall root-mean-square deviation between the structures ranging from 0.38 to 0.50 Å^2^. The regions of the protein with the highest conformational diversity are: the loop following α-helix 2 (58–61), part of the active site loop (216–228), and the loop between β-strands 13 and 14 (296–303). Interestingly, the latter two loops host residues belonging to the active site entrance (Figure 5A).

## Quaternary structure

hDAAO is always present as a head-to-head dimer, differently to other known DAAOs: the enzyme from rat is monomeric while the one from pig is a dimer or tetramer, depending on protein concentration (Molla et al., 2006; Pollegioni et al., 2007; Frattini et al., 2011). The apical surface of the SBD plays a central role in the monomer-monomer interaction together with the short α-helices 4 (83–88) and 6 (118–123) and the terminal residues of α-helix 10 (269–271). This region represents 9.8% of the overall solvent, accessible surface of the protomer (~1,523 Å^2^ as estimated using the PDBePISA tool) (Krissinel and Henrick, 2007). The substitution frequency at the dimer interface between hDAAO and pkDAAO (33% of the residues) is more than double that of the overall substitution frequency of the protein (15% of the residues). As a consequence, the charge distribution at the interface region of the human enzyme is different than other mammalian DAAOs: in hDAAO this surface is mainly negatively charged while the corresponding region in pkDAAO is slightly positively charged (Kawazoe et al., 2006). Interestingly, comparison of the dimerization interface between the dimeric hDAAO and the monomeric rat DAAO shows that even the substitution of only three residues in this region results in a different oligomeric state (Frattini et al., 2011). In particular, the substitution of Ser119 of rat DAAO with an arginine (Arg120) in hDAAO allows formation of additional interactions (one salt bridge and three H-bonds), which significantly contribute to stabilizing the monomer-monomer interface of the dimer. Importantly, and differently from all other flavoprotein oxidases, hDAAO retains its dimeric structure even in the apoprotein form. This suggests that the alteration in tertiary structure following removal of the FAD cofactor does not affect the interface surface (Molla et al., 2006).

## Cofactor binding

The FAD cofactor is embedded in the core of the FBD of the flavoprotein in an elongated conformation, forming a complex network of non-covalent interactions with the protein. A peculiar feature of the human enzyme is the low affinity for the cofactor: the K_d_ for FAD is 8 μM, a value ~40-fold (0.22 μM) and ~27-fold (0.30 μM) higher than that of porcine and rat DAAO, respectively (Molla et al., 2006; Frattini et al., 2011). Similar to what is observed in porcine DAAO, the affinity for the cofactor is increased ~30-fold by the presence of an active site ligand (Molla et al., 2006). The rational of the low affinity for the cofactor is still elusive but it could be due to the conformation of a small hydrophobic stretch next to FAD *si*-face of the isoalloxazine moiety (residues 47–51). In hDAAO, the main chain N of Ala49 cannot establish a H-bond with the N(5) atom of FAD, which is at a distance of 3.9 Å. In the porcine enzyme, this distance is reduced to 3.0 Å, allowing formation of a high-energy H-bond and thus increasing the strength of the interaction between the protein and the cofactor (Kawazoe et al., 2006).

The thermal stability of hDAAO (T_m_ = 51.8) (determined following the changes in protein fluorescence) is slightly higher than that of the ortholog from yeast (T_m_ = 46.4°C) (Pollegioni et al., 2003; Caldinelli et al., 2010). The large number of non-covalent interactions between the apoprotein and the cofactor contributes to the thermal stability of the enzyme: an increase in T_m_ of 1.6°C was evident following formation of the holoprotein (Caldinelli et al., 2010). Interestingly, the presence of a ligand at the active site of the enzyme also increases the stability of hDAAO; as an example, in the presence of trifluoro-D-alanine the T_m_ is 55.7°C (Table 2; Caldinelli et al., 2010).

**Table 2:** Main functional and structural properties of human D-amino acid oxidase.

**ENZYME PROPERTIES**			
Length	347 residues		
MW (monomer)	39,474 Da (apoprotein) 40,260 Da (holoenzyme)		
Quaternary structure^1^	Dimeric (both holo- and apoprotein)		
FAD affinity^1^	8.0 ± 0.2 μM (free enzyme) 3 ± 1 μM (in complex with benzoate)		
T_m_^2^	50.2°C (Apoprotein) 51.8°C (Holoenzyme) 55.7°C (Holoenzyme + CF_3_-D-Ala)		
**KINETIC CONSTANTS^1^**			
	**D-Ser**	**D-Ala**	
k_cat_ (s^−1^)	6.3 ± 1.4	14.7 ± 0.7	
k_red_ (s^−1^)	117 ± 6	180 ± 20	
**APPARENT KINETIC PARAMETERS**			
**Substrate**	**k_cat_ (s^−1^)**	**K_m_ (mM)**	**k_cat_ / K_m_ (s^−1^ mM)**
D-DOPA^3^	21.7	1.5	14.5
D-Tyr^3^	14.8	1.1	13.4
D-Ser	3.0^1^	7.5^1^	0.4^1^
	4.0^3^	3.9^3^	1.0^3^
D-Phe^3^	15.5	1.2	12.9
D-Pro^1^	10.2	8.5	1.2
D-Ala^1^	5.2	1.3	4.0
D-Asp^1^	~6.7	~2,000	<0.01
Gly^1^	0.9	180	<0.01

1 *Molla et al., 2006*.

2 *Caldinelli et al., 2010*.

3 *Kawazoe et al., 2007b*.

Apparent kinetic parameters were determined at 0.25 mM O_2._

## Architecture of the active site

The active site of hDAAO is formed by a cavity of ~220 Å^3^ in the SBD of the protein (Figure 5B). The α-carbon of the substrate amino acid and its carboxylic and amino substituents bind above the *re*-side of the isoalloxazine moiety, which forms the floor of the active site. The residue that mainly contributes to the binding energy of the substrate is Arg283, which forms a bidentate electrostatic interaction with the negatively charged α-COOH group of the amino acid. The α-COOH of the substrate is also connected through a H-bond to the hydroxyl of Tyr228. Arg283 and Tyr228 show a very high degree of evolutionary conservation based on the ConSurf analysis (Landau et al., 2005). Even a conservative mutation of Arg283 (e.g., to Lys), which is conserved in all known DAAOs, results in a dramatic drop in enzymatic activity (as reported for the yeast enzyme) (Molla et al., 2000). The α-NH_2_ of the substrate forms two H-bonds: one with the main chain oxygen of Gly313 (which, in homologous proteins, can be replaced by a small residue such as alanine or serine) and one with the oxygen of the C(4) = O group of FAD (Kawazoe et al., 2007b). In the structure of the yeast DAAO, the α-NH_2_ is H-bonded to a crystallographic water molecule (Umhau et al., 2000): such a solvent molecule is not observed in the structure of the human enzyme. As there is enough space in the active site to accommodate such a solvent molecule, its presence cannot be excluded. The substrate side chain is located in a mostly apolar cavity toward the active-site entrance, which is lined by the residues Leu51, Gln53, Leu215, and Ile230; this region is called “substrate specificity pocket.” Tyr224, which belongs to the flexible active-site loop, is able to form π-π stacking interactions with aromatic ligands (e.g., benzoate) (Kawazoe et al., 2006). Residues belonging to the substrate specificity pocket and Tyr224 show a medium degree of evolutionary conservation. The less conserved active-site residue in the family of amino acid oxidases is Leu215, which is the key residue for determining the substrate specificity (Sacchi et al., 2002). The fundamental role of active-site residues for binding substrates or inhibitors has been confirmed recently by using structural computational approaches (Wichapong et al., 2014).

## Kinetic properties and substrate preference

The kinetic and catalytic mechanism of DAAO has been described in detail using the yeast enzyme as a model (Pollegioni et al., 1993; Umhau et al., 2000). hDAAO catalyzes the oxidation of D-amino acids following a ternary-complex mechanism (Molla et al., 2006); the substrate dehydrogenation proceeds by directly transferring the hydride of the α-hydrogen from the α-carbon of the amino acid to the flavin N(5) (Figure 1B). These atoms are 3.6 Å apart in the complex of hDAAO with the product imino serine, but in the actual Michaelis complex (i.e., in the presence of D-Ser in the active site), this distance is probably shorter (~3.2 Å) due to the sp3 geometry of the α-carbon of the amino acid (Figure 5B). The reductive half-reaction is very fast (117 ± 6 s^−1^ on D-Ser) but the overall turnover is much smaller (6.3 ± 1.4 s^−1^) because the rate-limiting step of the reaction is release of the product (Molla et al., 2006; Table 2). At optimal pH (>8.0), the α-NH_2_ group of the substrate is neutral, but, at pH < 8.0, the substrate binds in the zwitterionic form and a proton must be simultaneously removed from the α-${\text{NH}}_{3}^{+}$ group of the substrate during the hydride transfer (Umhau et al., 2000). It has been proposed that, in yeast DAAO, the primary acceptor of the proton from the α-${\text{NH}}_{3}^{+}$ group is the hydroxyl of the side chain of Ser335 (Boselli et al., 2004). In the mammalian enzyme, where Ser335 is replaced by Gly313, an active-site water molecule (placed at a H-bond distance from the α-${\text{NH}}_{3}^{+}$) might play the same role, which could represent the first member of a proton relay chain between the substrate and the bulk solvent (Figures 1, 5; Boselli et al., 2004). Following hydride transfer, the negative charge formed on the reduced flavin is stabilized by the positive charge generated on the imino group of the product.

The strict preference of hDAAO for the D-enantiomer of the substrates can be explained by the four-location model for enantioselectivity proposed in 2000 by (Mesecar and Koshland, 2000). According to this model, the D-enantiomer of the amino acid forms three binding interactions: (i) between the α-COOH, (ii) the α-NH_2_ and (iii) the side chain and several residues of the hDAAO active site. In addition, the correct binding generates a “functional direction” represented by the alignment of the α-H of the amino acid and the N(5) of the flavin.

**Figure 6:**
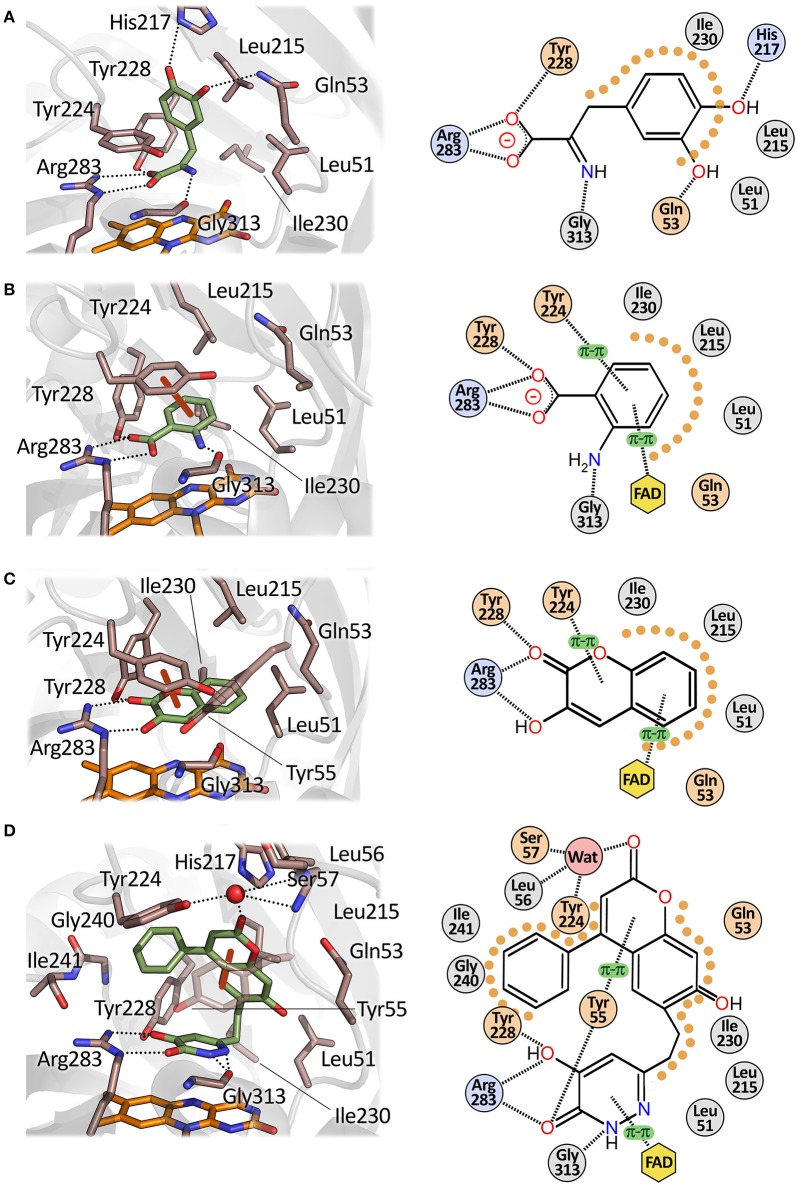
Mode of binding of hDAAO ligands. Human DAAO in complex with **(A)** imino-DOPA, PDB code 2E82; **(B)** o-Aminobenzoate, PDB code 2E4A; **(C)** 3-hydroxy-2H-chromen-2-one, PDB code 3ZNP; **(D)** 3-(7-hydroxy-2-oxo-4-phenyl-2H-chromen-6-yl)propanoic acid, PDB code 4QFD. Left: detail of the active site. The ligand is in green and the FAD in orange. Dotted lines represent H-bonds and thick brown lines represent π-π interactions. Right: schematic view of ligand-protein interactions. Dotted lines represent H-bonds and π-π interactions. Pale orange dots represent hydrophobic contacts.

The apparent kinetic parameters of hDAAO are similar to those of its homolog from pig kidney: as an example, the apparent k_cat_ and K_m_ for D-Ser of pkDAAO are 3.0 s^−1^ and 3.3 mM, respectively, while the corresponding values for the human enzyme are 3.1 s^−1^ and 7.5 mM, respectively (Molla et al., 2006; Setoyama et al., 2006). The human enzyme is active preferentially on bulky aromatic substrates (e.g., D-DOPA, D-Tyr, D-Phe, and D-Trp), followed by small uncharged D-amino acids (i.e., D-Ser, D-Ala and D-Pro). The very low K_m_ for 3,4-dihydroxy-phenylalanine (D-DOPA) is due to the formation of two additional H-bonds between the two hydroxyls of the side chain of the substrate and His217 and Gln53 (Figure 6A; Kawazoe et al., 2007b). Interestingly, DAAO activity was also detected in dopaminergic neurons of the human nigrostriatal system, suggesting that D-DOPA (a precursor of dopamine, norepinephrine, and epinephrine) could represent the physiological substrate of hDAAO in this region of the CNS (Wu et al., 2006; Sasabe et al., 2014).

The wide substrate promiscuity of the mammalian enzyme is due to its ability to bind the side chain of bulky substrates at the entrance of the active site, thanks to the plasticity of the active-site loop 217–227 (active site lid). The aromatic side chain of Tyr224, which can assume different orientations depending on the ligand bound at the active site of the protein, plays a key role (Kawazoe et al., 2007a). The plasticity of the active-site loop, extensively studied in the porcine enzyme (Todone et al., 1997; Vanoni et al., 1997), was confirmed by analyzing the complexes between hDAAO and bulky allosteric inhibitors (Terry-Lorenzo et al., 2014; Figure 6).

Very small or negatively charged amino acids such as Gly or D-Asp are poor hDAAO substrates because of a very high K_m_ (Molla et al., 2006; Table 2). Consequently, D-Asp, the second most abundant D-amino acid in mammalian CNS, is oxidized by the paralogue enzyme DASPO (Katane and Homma, 2010).

## Inhibitors of human DAAO

From the pathological point of view, a decreased local concentration of D-serine, which is an N-methyl-D-aspartate type glutamate receptor coagonist, has been correlated with hypofunction of the N-methyl-D-aspartate-mediated neurotransmission, which could result in severe CNS disorders (e.g., schizophrenia) (Panatier et al., 2006; Coyle, 2016). Two different strategies to restore the physiological concentration of D-Ser have been proposed. The first one consists in the oral administration of D-Ser (Kantrowitz et al., 2015); however, this approach is limited by the high dose that is required and the potential nephrotoxicity of a D-Ser excess (Orozco-Ibarra et al., 2007). The second one is represented by slowing down D-Ser degradation and inhibiting the DAAO activity (the main enzyme involved in the catabolism of this compound in CNS) (Sacchi et al., 2013). This latter strategy has been demonstrated to improve cognition and learning functions (Hopkins et al., 2013a). Several academic and industrial research groups focused on identifying hDAAO inhibitors (as potential pharmaceutical drugs) by combining structural bioinformatics, combinatorial chemistry, and high-throughput approaches. According to the BindingDB (searched using the UniProt code P14920), more than 500 compounds able to inhibit hDAAO activity *in vitro* and/or *in vivo* have been identified in the last 10 years (Gilson et al., 2016). Although the actual clinical relevance of most of these compounds is still to be fully evaluated, these molecules represent a valuable source of information about the structural determinants involved in substrate recognition and binding in the human flavoprotein. As a matter of fact, almost all of these compounds bind at the active site of hDAAO and act as competitive inhibitors with the substrate. In most cases, *in vitro* affinity can be easily calculated because binding of the inhibitors at the active site of the enzymes causes perturbation of the visible absorbance spectrum of the flavin (Molla et al., 2006; Katane et al., 2013).

Despite the large number of different potential hDAAO inhibitors, they consist of a limited number of different pharmacophores. In analogy with the substrate, the chemical structure of the inhibitors can be divided into two main portions: the first one is the “planar” part which forms specific interactions (electrostatic, H-bond, or hydrophobic interactions) with the core of the active site of the enzyme (i.e., the residues that are close to the flavin isoalloxazine moiety). The core of the “planar” portion of almost all inhibitors is formed by one or two fused rings, one of which can be aromatic. The ring(s) must have at least a negatively charged carboxylic group as a substituent or a bioisostere of this group that is able to form a bidentate H-bond and interact with the Arg283 of the active site. The second portion of the inhibitor corresponds to the substrate side chain: this region can form additional interactions with residues of the substrate specificity pocket(s) of the active site and/or of its entrance, depending on the size and chemical features of the inhibitor.

### Classical inhibitors

Single-ring ligands such as benzoate and anthranilate represent classical inhibitors of DAAOs (Sacchi et al., 2012; Katane et al., 2013). The carboxylic group of these compounds interacts with Arg283 while the aromatic ring forms π-π staking interactions with the side chain of Tyr224 at the active site of the protein (Figure 6B). The affinity constants for these compounds are in the μM range (Table 3).

**Table 3:** Selected of hDAAO inhibitors.

**Compound**	**Compound**	**M.W. (Daltons)[Atoms]**	**K_i_ (nM)**	**IC_50_ (nM)**	**IC_50_ Method**	**References**
1	Benzoate	122.12 [9]	7,000	N.D.	N.A.	Molla et al., 2006
3	Anthranilate	137.14 [10]	N.D.	N.D.	N.A.	Kawazoe et al., 2007a
16	5-Methylpyrazole- 3-carboxylic acid	126.11 [9]	390	910	Coupled (*o*-dianisidine), pH 9.0 10 mM D-Ser	Adage et al., 2008
			417^1^	473 ± 180	Amplex UltraRed, pH 7.4 [D-Ser] = 1 mM	Lange et al., 2011
5	4H-Furo[3,2-b]pyrrole-5-carboxylic acid	151.12 [11]	60.4^1^	141 ± 73	Amplex red Assayed in CHO cells transfected with DAAO, pH 8.2 [D-Ser] = 10 mM	Sparey et al., 2008
			9^1^	9 ± 2	Amplex Red, pH 8.5 [D-Ser] = 0.2 mM	Duplantier et al., 2009
			15^1^	17.2 ± 10.5	Amplex UltraRed, pH 7.4 [D-Ser] = 1 mM	Lange et al., 2011
7	4H-Thieno[3,2-b]pyrole-5-carboxylic acid	167.19 [11]	3.5+0.2	5.0 ± 0.8	Amplex Red, pH 7.4 [D-Ser] = 5 mM	Hopkins et al., 2013a
			61.7^1^	144.6 ± 16.1	Amplex red Assayed in CHO cells transfected with DAAO, pH 8.2 [D-Ser] = 10 mM	Smith et al., 2009
			10.5^1^	11.9 ± 6.3	Amplex UltraRed, pH 7.4 [D-Ser] = 1 mM	Lange et al., 2011
17	5-Chlorobenzo[d]isoxazol-3-ol (CBIO)	167.59 [11]	100	188	Not available	Ferraris et al., 2008
			15.0^1^	17.29 ± 8.8	Amplex UltraRed, pH 7.4 [D-Ser] = 1 mM	Lange et al., 2011
18	5-Chloro-6-fluoro-3-hydroxy- 4a,5-dihydro-1,8-naphthyridin-2(1H)-one	214.58 [14]	2.3^1^	3	Amplex Red, pH 8.5 [D-Ser] = 0.2 mM	Duplantier et al., 2009
8	3-Hydroxy-2H-chromen-2-one	162.14 [12]	13 ± 1.4	34.7	Amplex Red, pH 7.4 [D-Ser] = 5 mM	Hopkins et al., 2013a
			156 ± 27^1^	440 ± 29	2 4-Dinitrophenylhydrazine, pH 8.3 [D-Ser] = N.A.	Katane et al., 2013
19	5-Hydroxy-2-hydroxymethyl-4H-pyran-4-one (kojic acid derivative)	319.2 [19]	60^1^	100	Coupled (o-phenylenediamine), pH 8.2 [D-Ser] = 5 mM	Raje et al., 2013
20	6-Hydroxy-2-(naphthalen-1-ylmethyl)-1,2,4-triazine-3,5(2H,4H)-dione	269.26 [20]	60	50 ± 10	Coupled (o-phenylenediamine), pH 8.5 [D-Ser] = 5 mM	Hin et al., 2015
21	3-((6-Fluoronaphthalen-2-yl) methylthio)-6-hydroxy-1,2,4-triazin-5(2H)-one	300.36 [21]	18	30	Coupled (o-phenylenediamine), pH 8.5 [D-Ser] = 5 mM	Hin et al., 2016
14	4-Hydroxy-6-[2-(7-hydroxy-2-oxo-4-phenyl-2H-chromen-6-yl)ethyl]pyridazin-3(2H)-one	376.36 [28]	9.6 ± 1.0	20 ± 5	Amplex Red, pH 7.4 [D-Ser] = 5 mM	Terry-Lorenzo et al., 2014
22	2-(2,5-Dimethylphenyl)-6-fluorobenzo[d]isothiazol-3(2H)-one	273.32 [19]	N.D.	360 ± 140	Amplex Red, pH 7.4 [D-Ser] = 5 mM	Terry-Lorenzo et al., 2015
23	Substitute triazole	497.5 [36]	500,000^2^	N.A.	N.A.	Toguchi et al., 2016

*N.D., not determined, N.A., not applicable*.

1 *Estimated using Cheng-Prusoff formula*.

2 *Inhibition vs. benzoa*.

**Figure 7:**
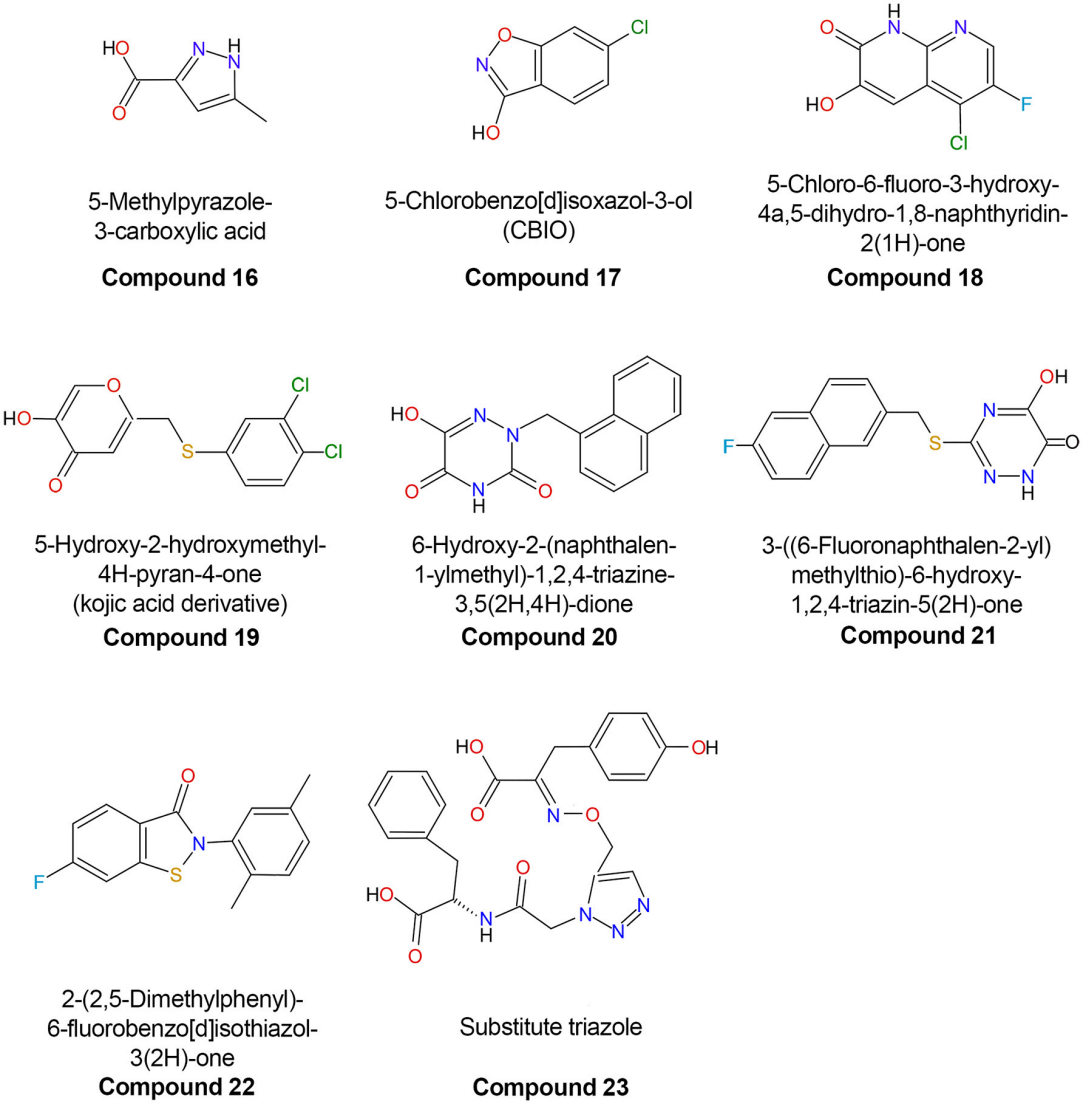
Chemical structures of human D-amino acid oxidase inhibitors reported in Table 3.

*IC_50_ values were determined under different experimental conditions and D-Ser concentrations. Structures of ligands are reported in Figures 4, 7*.

Compounds in which one or more carbon atoms of the ring(s) are substituted by O, N, or S represent improved variants of classical inhibitors. An example of this kind of compound is represented by 5-methylpyrazole-3-carboxylic acid (**compound 16**), in which two carbon atoms of the ring are substituted by nitrogen (Figure 7; Table 3). The considerable affinity of hDAAO for this compound (K_i_ = 0.39 μM) is due to a better network of H-bonds formed at the active site. *In vivo* and *ex vivo* studies demonstrated that this compound was able to cross the blood-brain barrier (BBB) in rats, increasing the level of D-Ser in selected regions of the brain and reversing the pharmacological effects induced by phencyclidine (Adage et al., 2008).

### Second-generation inhibitors

The second generation of hDAAO inhibitors is comprised of compounds formed by two substituted, heterocyclically fused rings. The larger planar core of such ligands (which possess on average an ~27% increased volume in comparison with classical single-ring inhibitors) allows optimization of the van der Waals interactions at the active site. The formation of additional H-bond interactions with the enzyme residues is granted by the presence of one or more heteroatoms in the ring system. The first member of this class to be discovered was a fused pyrrole carboxylic acid composed of two 5-member rings **(compound 5)** possessing an IC_50_ of 141 nM. Administration of **compound 5** to rats (at a dosage of 150 mg/kg) resulted in a more than 2-fold increase in D-Ser concentration in plasma and in an *ex vivo* hDAAO inhibition of the enzymatic activity in kidney and cerebellum (Sparey et al., 2008). In 2013, another inhibitor possessing a very similar structure was discovered: 4H-thieno[3,2-b]pyrole-5-carboxylic acid in which the O atom of the furan moiety was replaced by a S atom (**compound 7**, Table 3). This moiety is placed in the hydrophobic pocket of the active site in close contact with the side chains of the apolar amino acids Leu51, Leu215, and Ile230 and it interacts via π-stacking interactions with the FAD cofactor. This compound showed an inhibitory effect (K_i_ = 3.5 nM) 3 orders of magnitude higher than benzoate. In accordance with its very low IC_50_, administration of **compound 7** to mice increased D-serine levels even at low dosage (12 mg/kg every 2 h) (Hopkins et al., 2013b).

Increasing the size of the planar core of the inhibitor by substituting one or two 5-member ring(s) with one or two 6-member ring(s) allowed the formation of better hydrophobic van der Waals interactions with the apolar residues of the enzyme active site. Compounds formed by two 6-member substituted rings are the most effective second-generation inhibitors (Duplantier et al., 2009; Hopkins et al., 2013b; Katane et al., 2013). In these compounds, the carboxylic group of the ligand is usually replaced by a bioisosteric group which retains the ability to form two H-bonds with Arg283 but, at the same time, possesses an increased ability to penetrate the BBB because of the absence of a negative charge (Duplantier et al., 2009). Removal of the negatively charged carboxylic group does not grant an easy passage through the BBB as shown by 5-chlorobenzo[d]isoxazol-3-ol (CBIO, **compound 17**), which did not enhance D-serine levels in the brain (Ferraris et al., 2008).

A series of compounds derived from 3-hydroxyquinolin-2(1H)-one have been tested as hDAAO inhibitors (Duplantier et al., 2009). Compounds possessing substituents on the ring facing Arg283 showed very poor binding because of steric clashes. On the other hand, the presence of halogen substituents (fluorine or chlorine) on the second ring slightly increased the potency of the inhibitor. The best compound of this series (**compound 18**) has an IC_50_ of 3 nM which, at present, represents the lowest value among the reported hDAAO inhibitors, even if it was determined at a very low D-Ser concentration (0.2 mM, Table 3). Mice treated with 10 mg/kg of **compound 18** showed D-Ser levels in cerebellum up to 6-fold higher than in the control 4 h after administration (Duplantier et al., 2009). A few years later, the research groups led by Hopkins and Katane independently identified a similar compound (3-hydroxy-2H-chromen-2-one, **compound 8)** in which the cyclic nitrogen was substituted by an oxygen atom (Table 3) (Hopkins et al., 2013b; Katane et al., 2013). Analysis of the complex between **compound 8** and hDAAO showed that the side chain of Tyr224 was shifted 1.5 Å toward the planar rings of the ligand, forming an additional π-π staking interaction while Tyr55 side chain took the place originally occupied by Tyr224 (Figure 6C; Hopkins et al., 2013b). **Compound 8** was able to efficiently cross both cell and peroxisomal membrane of human embryonic kidney 293 cells to reach and effectively inhibit hDAAO (Katane et al., 2013).

### Third-generation inhibitors

Analysis of the mode of binding of imino-DOPA at the active site of hDAAO revealed the existence of an additional second binding pocket (named active site “subpocket”) at the entrance of the active site that can harbor the cyclic moieties of the side chain of bulky and flexible ligands (Figure 6A; Kawazoe et al., 2007b; Hondo et al., 2013). Different research groups took advantage of the additional interactions of the ligand with the residues located at the entrance of the active site to design novel third-generation inhibitors. The first example of compounds that exploit this different mode of interaction with hDAAO are the kojic acid derivatives (e.g., **compound 19**) (Raje et al., 2013). This class of compounds, in which a substituted O-phenyl derivative was connected to kojic acid by a 2-atom linker, showed an IC_50_ in the range of hundreds of nM (Table 3). Docking analysis of **compound 19** to the active site of different experimental structures of hDAAO supported the hypothesis that the phenylthiomethyl group of the inhibitor is bound in the subpocket created by the conformational change of Tyr224 (Raje et al., 2013). The same research group identified a series of inhibitors with a similar steric hindrance [6-hydroxy-1,2,4-triazine-3,5(2H,4H)-dione derivates and 3-substituted 5-hydroxy-1,2,4-triazin-6(1H)-one derivatives], but possessing a lower IC_50_ (corresponding to 30 nM for **compound 21**, the best one). These inhibitors also showed an increased resistance to metabolic degradation and better oral availability (Table 3) (Hin et al., 2015, 2016).

Using a computational chemistry approach and structural bioinformatic prescreening followed by high-throughput screening of the 1016 best-scoring compounds (Terry-Lorenzo et al., 2014) **compound 14** was identified (IC_50_ = 20 ± 5 nM) as an efficient inhibitor: in this compound, the carboxylic acid moiety of canonical hDAAO inhibitors was replaced by a hydroxyl-pyridazinone group bioisostere. This moiety forms a total of five hydrogen bonds with active-site residues: two with the guanidinium group of Arg283, one with the Tyr228-OH, one with the Tyr55-OH, and one with the backbone carbonyl of Gly313 (Figure 6D). The remaining portion of the molecule is formed by a phenyl-substituted coumarin heterocycle linked to the planar core of the inhibitor by a 2-carbon flexible linker. The phenyl group (at the position 4 of the coumarin group) is located in the hydrophobic active-site subpocket formed by residues Ser226, Tyr 224, Tyr228, and Gly240. These residues, with the exception of Gly240, belong to the flexible active-side lid. The conformational change of this loop, and in particular of Tyr224 that is moved 8 Å away from the active site, allows the formation of the novel binding subpocket at the active-site entrance. The conformational change of Tyr224 is followed by a concomitant movement of the side chain of Tyr55 which is shifted 3.8 Å toward the ligand, resulting in the formation of two additional interactions: a H-bond with the carbonyl oxygen of the ligand bioisosteric group and a π-π-staking interaction with the aromatic ring of the coumarin heterocycle. These large numbers of non-covalent interactions allow **compound 14** to form a very sticky interaction with the enzyme with a dissociation constant (k_off_) of 0.015 ± 0.03 min^−1^ (Terry-Lorenzo et al., 2014). The observation of the alternative (open) conformation of the active-site loop in the 3D structure of hDAAO in complex with **compound 14** confirms the flexibility of the active-site lid previously predicted only from the structural investigation of the porcine enzyme (Todone et al., 1997).

The mode of binding of **compounds 10–15** suggests that the active site of hDAAO can be divided into two distinct regions that possess a different structural flexibility. The core of the active site, close to the *re*-side of the isoalloxazine moiety of FAD, appears to be very rigid; this property is required to bind the substrate in the precise orientation to favor hydride transfer during the chemical step of catalysis (Umhau et al., 2000). On the other hand, residues forming the upper part of the active site and its entrance easily alter their conformation to conform to the shape of the ligand and represent the main tool for substrate promiscuity of hDAAO. The high affinity of **compound 14** (K_i_ = 9.6 nM) suggests that the interactions formed at the substrate subpocket can provide more energy than the one deriving from π-π stacking interactions with the side chain of Tyr224 or the FAD isoalloxazine. Compounds designed to maximize these kinds of interactions, to the detriment of polar interactions at the core of the active site, might make it possible to design very potent novel inhibitors possessing structural properties consistent with Lipinski's “rule of five” (Lipinski et al., 2001; Terry-Lorenzo et al., 2014).

### Novel-generation inhibitors

A very recent class of hDAAO inhibitors has been identified by innovative high-throughput screening and the work performed by Pollegioni's lab on pLG72 (Terry-Lorenzo et al., 2015). *In vivo*, the interaction between hDAAO and its regulatory partner pLG72 could influence the ability of inhibitors to bind the human flavoenzyme (e.g., by shielding the binding site or by inducing conformational change) (Sacchi et al., 2008; Birolo et al., 2016). A high-throughput screening was set up to assay the inhibitory effect of compounds in the absence and in the presence of 0.7 μM pLG72, a concentration corresponding to the IC_20_ of pLG72; at this concentration of pLG72, the protein is in a dynamic state of binding equilibrium with hDAAO. Starting from a library of ~150,000 compounds, 102 inhibitors were identified and classified into three groups: 9 compounds which were more potent in the presence of pLG72 (Class A compounds), 86 compounds whose potency was independent of the presence or absence of pLG72 (Class B compounds), and 7 compound which were 5- to 10-fold less potent when pLG72 was present (Class C compounds). All these compounds possess a 2-phenyl-2,3-dihydro-1,2-benzothiazol-3-one (also named ebsulfur) substructure.

**Compound 22** (Table 3) represents the inhibitor belonging to class C compounds that was most thoroughly characterized. Interestingly, it shows a mixed-inhibition mechanism consistent with a FAD-competitive behavior. Importantly, the inhibitory effect was evident only under oxidizing conditions; as a matter of fact, in the presence of 5 mM glutathione, **compound 22** was completely impotent as a hDAAO inhibitor. **Compound 22** did not show a detectable dissociation from hDAAO in jump-dilution experiments and the hDAAO activity was not recovered even 24 h after dilution. The enzymatic activity of the human flavoprotein could be fully recovered only by adding a reducing compound to the assay mixture. Since the isothiazolone group of **compound 22** can form covalent S-S thiol bonds with cysteines, the authors reported that, under oxidizing conditions, this inhibitor formed a S-S thiol bond with each of the 5 cysteines of hDAAO (Cys18, Cys181, Cys263, Cys264, and Cys322). This non-specific cysteine-binding behavior is apparently incompatible with the strict inhibition specificity for hDAAO of this compound, which does not inhibit flavoprotein oxidases such as the human DASPO or glucose oxidase. On the other hand, it must be pointed out that, in flavoproteins, cysteines are usually buried in the protein core (the cysteines of hDAAO show <15% of solvent-accessible surface) and are accessible to thiol-crosslinking agents only when the protein is partially unfolded (Terry-Lorenzo et al., 2015). Human DAAO represents an exception among the mammalian flavooxidases as it has a very low affinity for the FAD cofactor; as a consequence, *in vivo* hDAAO exists in an equilibrium between holoprotein and apoprotein (Molla et al., 2006). This latter form is characterized by a less structured and compact conformation, allowing increased accessibility to its cysteines (Caldinelli et al., 2004, 2009). According to this model, binding of pLG72 to hDAAO could shield the flavoprotein cysteines or could cause a conformational change of the flavoenzyme that could prevent formation of the disulfide bond (Terry-Lorenzo et al., 2015).

Recently, the “click chemistry” approach was employed to design innovative hDAAO inhibitors. In this approach, the enzyme active site provides the template for the *in situ* target-guided synthesis of the inhibitor. Based on the mode of binding of imino-DOPA at the active site of hDAAO, different pairs of reactive compounds (organic azides and alkynes) were screened for the ability to form a covalent product (a triazole) at the active site of the human enzyme possessing a higher inhibitory potency than the starting reagents. **Compound 23** possesses a K_i_ (in a competition assay vs. the classical inhibitor benzoate) of 500 μM (Toguchi et al., 2016). Although, this value is significantly higher than for a number of known inhibitors, this strategy could represent a promising strategy for identifying novel hDAAO inhibitors.

### hDAAO inhibitors that do not compete with the substrate

Two inhibitors have been identified that compete with the cofactor FAD for binding to hDAAO: ADP (IC_50_ = 580 ± 80 μM) and CPZ (K_d_ = 5 μM; K_i_ = 0.7 mM) (Sacchi et al., 2008; Terry-Lorenzo et al., 2014). Adding CPZ to a solution containing hDAAO holoprotein induces a slow, time-dependent replacement of the physiological FAD cofactor by the drug, which progressively inactivates the enzyme (Sacchi et al., 2008). As a matter of fact, the presence of 0.1 mM CPZ increases the sensitivity of hDAAO holoenzyme to trypsinolysis: a 10-fold increase in the rate constant of the second phase of proteolytic cleavage (k_obs2_ = 6 × 10^−2^ min^−1^), which is related to the slow FAD dissociation from holoenzyme form, was observed. In the presence of 0.1 mM CPZ and 40 μM FAD, the thermal stability of hDAAO also decreased from 51.8 to 48°C (Caldinelli et al., 2010). Although CPZ binds hDAAO with an affinity similar to that of FAD (8 × 10^−6^ and 5 × 10^−6^ M for FAD and CPZ, respectively), the comparison of the near-UV CD spectra suggests that the tertiary CPZ-hDAAO structure is different than that of the FAD-hDAAO holoprotein. Indeed, CPZ favors a protein conformation resembling that of the apoprotein which possesses a less stable and more denaturation-prone tertiary structure (Caldinelli et al., 2010). In the presence of white light, CPZ forms oligomers through a radical-induced mechanism. The oligomerized CPZ is still biochemically active since it is able to inhibit hDAAO activity, probably by inhibiting the formation of the active holoprotein form of the flavoenzyme (Iwana et al., 2008).

## Modulation of hDAAO properties by interaction with other proteins

At present, only three physical interactions between hDAAO and other proteins have been experimentally demonstrated. The first one is the interaction between hDAAO and the PTS1-receptor Pex5p (coded by the gene *PEX5*), a protein that plays an essential role in peroxisomal protein import and in the assembly of functional peroxisomes (Ghosh and Berg, 2010) and that interacts with hDAAO through the C-terminal PTS1-type peroxisomal-targeting signal (SKL-type tripeptide). The second hDAAO partner is pLG72, a primate-specific protein (153 residues, 18.1 kDa) with a very short half-life (t_1/2_ ~ 25–40 min) which was identified by using a yeast two-hybrid approach. This protein is coded by the gene *G72*, which has been associated with schizophrenia (Chumakov et al., 2002; Sacchi et al., 2008). Size-exclusion chromatography and surface plasmon resonance experiments demonstrated that pLG72 binds hDAAO with a dissociation constant of 0.08–0.53 μM (depending on the experimental conditions), yielding a ~200-kDa complex formed by two hDAAO homodimers and two pLG72 monomers (Sacchi et al., 2008; Caldinelli et al., 2010). The affinity of hDAAO for pLG72 is not altered by the presence of small ligands (e.g., FAD, CPZ, or D-Ser). The mode of interaction of the two proteins in the complex is still elusive. Recently, by applying low-resolution techniques (i.e., limited proteolysis couple to mass spectroscopy and cross-linking experiments) structural determinants of the proteins involved in the formation of the interface surface in the complex could be mapped. This study proposed a model of the hDAAO-pLG72 complex in which the N-terminal region of the protein plays an important role in forming the oligomerization interface (Birolo et al., 2016; Sacchi et al., 2017). Binding of pLG72 to hDAAO *in vitro* does not alter the kinetic parameters on D-Ser and the affinity for the FAD cofactor of hDAAO but rather increases the rate of hDAAO inactivation. This effect has been related to an alteration in the tertiary structure of hDAAO caused by pLG72 binding (Sacchi et al., 2008; Caldinelli et al., 2010). Interestingly, *in vivo*, the interaction between hDAAO and pLG72 dramatically alters the half-life of the flavoprotein; co-expression of pLG72 and hDAAO in U87 glial cells decreases the half-life of hDAAO from ~60 to ~6 h (Cappelletti et al., 2014). Ligands able to stabilize hDAAO-pLG72 complex could represent novel putative drugs to restore D-Ser concentration.

The third hDAAO interactor is the bassoon protein. Using a coimmunoprecipitation technique and mass spectrometry, Popiolek and collegues identified 24 putative DAAO-interacting proteins from rat cerebellum that were involved in the formation of presynaptic cytoskeletal protein matrix (“cytometrix assembled at the active zones”). The interaction between hDAAO and bassoon protein was also confirmed by coimmunoprecipitation assays on HEK293 cell extracts. The effect of such interactions is the apparent inhibition of DAAO enzymatic activity. The authors hypothesized that the ultimate goal of the hDAAO-bassoon interaction was similar to that between hDAAO and pLG72, which is the tight regulation of hDAAO activity to prevent excessive D-Ser depletion in the body of different CNS cell types or at the presynaptic active zone in neurons, in the case of pLG72 or bassoon protein, respectively (Popiolek et al., 2011).

## Conclusions

The huge efforts by public and corporate research laboratories to identify novel pharmaceutical drugs to fight schizophrenia symptoms identified hundreds of different hDAAO ligands which *in vitro* are able to inhibit the human flavooxidase with a potency up to 3 orders of magnitudes higher than classical inhibitors (e.g., benzoate, see Table 3). These compounds were also effective in inhibiting hDAAO activity *ex vivo* and/or *in vivo* as demonstrated by measuring residual hDAAO activity in cell lines and tissues from murine models. Unfortunately, none of the identified hDAAO compounds has yet been approved for schizophrenia treatment (or other diseases). The main drawbacks of these compounds are the poor bioavailability, high rate of clearance, and poor ability to cross the BBB. Consequently, the observed increase in D-Ser in the CNS observed after administration of such compounds should mainly be due to the systemic increase in the concentration of this D-amino acid caused by inhibiting kidney hDAAO (Hin et al., 2015). An additional drawback is the fact that the human and murine DAAOs show different biochemical properties and expression patterns. Consequently, the efficacy, pharmacokinetics, and pharmacodynamic properties of potential hDAAO inhibitors tested in rat, the “standard” animal model in pharmaceutical research, could be misleading (Frattini et al., 2011; Sasabe et al., 2014). As a matter of fact, the distribution of hDAAO activity between mouse and human CNS is different as, in the former, the activity of the enzyme is confined to the lower brain. This suggests that cells able to express DAAO may have migrated to the cerebral cortex in concert with the evolution of human forebrain (Sasabe et al., 2014).

Nevertheless, from a biochemical point of view, these compounds have become an invaluable source of information about the structure/function relationships in hDAAO. Since the “silver age” of the biochemical studies of flavoproteins (mid 1960s), modified flavins (used as spectral, chemical, or mechanistic probes or as photoaffinity labels) represented the main tool for investigating the structural and functional features of the active center of such enzymes (Massey and Hemmerich, 1980; Ghisla and Massey, 1986). This was facilitated by the ease with which the cofactor can be reversibly removed from this enzyme. Since 2006, hDAAO-competitive inhibitors have successfully replaced modified flavins in the biochemical investigation of this flavoprotein; they have enabled us to precisely define the roles of the active-site residues, identify novel binding subpockets at the active site, and investigate the conformational plasticity of the active-site loop. However, several properties of hDAAO are still elusive: the “actual” structural determinants responsible for the low affinity for the cofactor, the mode of interaction with protein partners such as pLG72, the subcellular localization and the function of extraperoxisomal hDAAO, and the regulation *in vivo* of hDAAO activity and turnover.

## Author contributions

The author confirms being the sole contributor of this work and approved it for publication.

### Conflict of interest statement

The author declares that the research was conducted in the absence of any commercial or financial relationships that could be construed as a potential conflict of interest.

## Abbreviations

CPZ: Chlorpromazine
BBB: blood-brain barrier
CBIO: 6-Chlorobenzo[d]isoxazol-3-ol
CNS: Central nervous system
DAAO: D-Amino acid oxidase
DASPO: D-aspartate oxidase
DOPA: 3,4-Dihydroxy-phenylalanine
hDAAO: Human D-amino acid oxidase
pkDAAO: Porcine kidney D-amino acid oxidase
PTS1: Peroxisomal targeting signal 1
SZ: schizophrenia
T_m_: Melting temperature.
